# Base-CP proteasome can serve as a platform for stepwise lid formation

**DOI:** 10.1042/BSR20140173

**Published:** 2015-05-19

**Authors:** Zanlin Yu, Nurit Livnat-Levanon, Oded Kleifeld, Wissam Mansour, Mark A. Nakasone, Carlos A. Castaneda, Emma K. Dixon, David Fushman, Noa Reis, Elah Pick, Michael H. Glickman

**Affiliations:** *Department of Biology, Technion–Israel Institute of Technology, 32000 Haifa, Israel; †Department of Biochemistry & Molecular Biology, Monash University, Clayton, VIC 3800, Australia; ‡Department of Biology and Environment, University of Haifa at Oranim, Tivon 36006, Israel; §Department of Chemistry and Biochemistry, Center for Biomolecular Structure and Organization, University of Maryland, College Park, MD 20742, U.S.A.

**Keywords:** 26S proteasome, 19S regulatory particle, 20S core particle, lid, base, MPN, PCI, rpn11-m1, CP, 20S core particle, DUB, deubiquitinase, polyUb, polyubiquitin, RP, 19S regulatory particle, TPP, trans proteomic pipeline, Ub, ubiquitin, WCE, whole cell extract, WT, wild-type

## Abstract

26S proteasome, a major regulatory protease in eukaryotes, consists of a 20S proteolytic core particle (CP) capped by a 19S regulatory particle (RP). The 19S RP is divisible into base and lid sub-complexes. Even within the lid, subunits have been demarcated into two modules: module 1 (Rpn5, Rpn6, Rpn8, Rpn9 and Rpn11), which interacts with both CP and base sub-complexes and module 2 (Rpn3, Rpn7, Rpn12 and Rpn15) that is attached mainly to module 1. We now show that suppression of *RPN11* expression halted lid assembly yet enabled the base and 20S CP to pre-assemble and form a base-CP. A key role for Regulatory particle non-ATPase 11 (Rpn11) in bridging lid module 1 and module 2 subunits together is inferred from observing defective proteasomes in *rpn11–m1*, a mutant expressing a truncated form of Rpn11 and displaying mitochondrial phenotypes. An incomplete lid made up of five module 1 subunits attached to base-CP was identified in proteasomes isolated from this mutant. Re-introducing the C-terminal portion of Rpn11 enabled recruitment of missing module 2 subunits. *In vitro*, module 1 was reconstituted stepwise, initiated by Rpn11–Rpn8 heterodimerization. Upon recruitment of Rpn6, the module 1 intermediate was competent to lock into base-CP and reconstitute an incomplete 26S proteasome. Thus, base-CP can serve as a platform for gradual incorporation of lid, along a proteasome assembly pathway. Identification of proteasome intermediates and reconstitution of minimal functional units should clarify aspects of the inner workings of this machine and how multiple catalytic processes are synchronized within the 26S proteasome holoenzymes.

## Abstract 2

Defective proteasome 19S regulatory particles (RPs) were identified in *rpn11f–m1*, a proteasomal mutant with mitochondrial phenotypes. The Rpn11 subunit initiates assembly of a five-subunit lid module competent to integrate into pre-assembled base-20S core particle (CP), with subsequent recruitment of remaining lid subunits.

## INTRODUCTION

Simultaneous processes of protein synthesis and degradation dictate the dynamics of the cellular proteome in eukaryotes [1]. Polyubiquitin (polyUb) modifications drive the destruction of a majority of cellular proteins by targeting them either to the lysosome or to a 2.5 MDa multi-catalytic protease, the 26S proteasome [2–4]. Structurally, the proteasome consists of a cylindrical proteolytic 20S core particle (CP) capped by a 19S regulatory particle (RP) at either ends [5,6]. By synchronizing polyUb recognition, deubiquitination, substrate unfolding and translocation, these 19S RP caps control proteolytic efficiency [7–15]. About 20 different subunits with distinguished structural motifs and dedicated functions make up the 19S RP [5,16–18]. Covering the entry port into the 20S CP, the base contains a ring of six AAA–ATPase subunits (Rpt1, Regulatory particle triple-A ATPase 1–Rpt6) and five additional subunits involved in anchoring or processing polyUb or ubiquitin (Ub)-like domains [Rpn1, Rpn2, Rpn10, Rpn13 and the transiently associated deubiquitinase (DUB) Ubp6–USP14, ubiquitin-specific processing 14] [3,19]. Nine subunits make up the lid, two of which Rpn8 (Regulatory particle non-ATPase 8) and Rpn11, belong to the Mpr1/Pad1 N-terminal (MPN)–JAB1/MPN/Mov34 Metalloprotease (JAMM) metalloprotease-related family [20], although only Rpn11 is a functional DUB [21,22]. CryoEM analysis of proteasome holoenzymes benefited from crystal structures of the MPN domains of Rpn11 and Rpn8, to localize them at the centre of the 19S RP in close contact to the RPT ATPases directly above the central pore [21,23–27]. DUB activity of Rpn11 is greatly enhanced when complexed into 26S proteasome holoenzymes [10,13,20,28,29]. The remaining lid subunits (Rpn3, Rpn5, Rpn6, Rpn7, Rpn9, Rpn12) form a horseshoe arrangement through their C-terminal PCI (porteasome, COP9 signalosome, eukaryotic initiation of translation factor 3) domains, whereas their more divergent N-terminal parts extend radially outwards [17,18,30]. Architecturally, lid subunits are arranged in two lobes: five interlocking subunits in module 1 (Rpn5, Rpn6, Rpn8, Rpn9 and Rpn11) and three subunits in module 2 (Rpn3, Rpn7 and Rpn12) are tethered via the non-essential Rpn15 (a.k.a Sem1) subunit [17,31–41]. No enzymatic information has been documented for lid subunits other than Rpn11. Apparently unrelated to its MPN^+^ enzymatic domain, the C-terminus of Rpn11 also plays a role in the stability of proteasomes and in mitochondrial integrity [42,43]. The importance of the C-terminal segment of Rpn11 and how it participates in proteasome assembly and stability is the focus of the current study.

Construction of a large multi-subunit machine such as the proteasome is expected to require high precision [44]. 20S CP biogenesis is an ordered stepwise process requiring five dedicated chaperones that have been described in detail [45,46]. At least four additional chaperones assist formation of the base as an independent sub-complex of the 19S RP [47–49]. Preassembled base may recruit lid subunits to generate a 19S RP precursor, which has been proposed to complex with 20S CP to form 26S proteasome holoenzyme [50]. Other observations, however, have noted that 20S CP may serve as a platform for layered assembly of 19S RP subunits [34,51]. Regardless of assembly pathway, 19S RP can detach from proteasome holoenzymes and re-attach un-aided by chaperones [48,52–55]. The lid too can detach and re-attach to base, the equilibrium of which is affected by various subunits such as Rpn10 or Sem1 [16,39,56–59].

Within the lid, module 2 subunits aided by Sem1 were proposed to assemble on to pre-assembled module 1 [38]. The final step was incorporation of Rpn12 marking the seal of proper lid [39,60]. Thus far, module 1 is the only proteasome sub-complex for which no assembly chaperone has been reported [39,61]. How does module 1 nucleate into a distinct sub-complex and what governs the association with the base on one side or module 2 on the other, were objectives of the present study. We show that both the MPN domain subunits, Rpn8 and Rpn11, play critical roles initiating lid assembly, without which lidless proteasomes were generated. More specifically, the C-terminal helix of Rpn11 linked module 2 to module 1 at the proteasome. Even in absence of this segment, module 1 retained its inherent DUB activity and was competent to bind base-CP both *in vitro* and *in vivo*. Loss of its C-terminus did substantially hamper ability of Rpn11 (incorporated on to module 1) and to recruit module 2. Proteasome specie consisting of module 1 base-CP has not been characterized before, yet we now find it abundant in rpn11–1 [42,43,55,62–64].

## EXPERIMENTAL

### Yeast strains

Table 1 contains a list of yeast strains used in the present study; the genetic background is based on BY4741:

**Table 1:** Yeast strains used in the present study The genetic background of the yeast strains used in the present study is based on BY4741

Number	Gene	Characteristics	Origin
MY58	WT	*his3ko1; leu2ko0; met15ko0; ura3ko0*	Euroscarf
MY1262	*tetO_2_–Rpn8*	*RPN8*::kanR*–*tetO_7_*–*TATA*–RPN8*	GE openbiosystems
MY1263	*tetO_2_–Rpn11*	*RPN11*::kanR*–*tetO_7_*–*TATA*–RPN11*	GE openbiosystems
MY1107	*rpn3–4*	*rpn3*::*rpn3–4–*TRP1	[65]
MY1070	*rpn5–1*	*rpn5*::*rpn5–1–*TRP1	[34]
MY1122	*rpn6–1*	*rpn6*::*rpn6–1*–URA3	[66]
MY1068	*rpn7–3*	*rpn7*::*rpn7–3*–URA3	[67]
MY1123	*rpn9ΔC*	*rpn9*::*rpn9ΔC*–LEU2	[68]
MY1119	*rpn12–1*	*rpn12*::*rpn12–1*–URA3	[65]
MY1268	*rpn11–m1*	*rpn11::rpn11–M1*	[42]
MY1284	*rpn8–1*	*rpn8::rpn8–1–LEU*	Present study

Abbreviation: WT, wild-type.

Table 2 contains a list of all plasmids used in the present study.

**Table 2:** List of plasmids used in the present study

Number	Gene	Vector	Origin
M1364	Rpn5, Rpn6, Rpn8, Rpn9, his6-Rpn11	petDuet	[17]
M1335	His_6_–Rpn11	pQE30	present study
M1388	Rpn8, His_6_–Rpn11	petDuet	present study
M1398	Rpn5, Rpn8, His_6_–Rpn11	petDuet	present study
M1397	Rpn5, Rpn8, Rpn9, His_6_–Rpn11	petDuet	present study
M1400	Rpn6, Rpn8, Rpn9, His_6_–Rpn11	petDuet	present study
M1386	Rpn5, Rpn6, Rpn8, His_6_–Rpn11	petDuet	present study
M1403	Rpn5, Rpn6, Rpn9, His_6_–Rpn11	petDuet	present study
M1109	Rpn11 C-terminus	pQE30	present study
M899	Rpn11 C-terminus	pRS425	[43]

The genetic background of the yeast strains used in the present study is based on BY4741

### Native gel and Western blotting of whole cell extract

Yeast whole cell extract (WCE) was prepared by glass beads vortexing in buffer A (25 mM Tris, pH 7.4, 5 mM MgCl_2_, 1 mM ATP 150 mM NaCl and 1 mM DTT) and cleared by centrifugation at 14000 ***g*** for 15 min. The soluble proteins were resolved using 4% native-PAGE, then visualized by LLVY-AMC peptidase activity assay during which the 0.1% SDS (w/v) was used in order to visualize the lower bands [16,69,70].

### Antibodies

The following antibodies were used to identify proteasome subunits: anti-Rpn1 and anti-Rpn2 [71]; anti-Rpt1 and anti-Rpt2 (present work); anti-Rpn11 [35], anti-Rpn12 (present work), anti-Rpn8 (present work) and anti-Rpn5 (gifts from Dan Finley).

### Gene silencing

Strains with suppressible proteasome genes, *tetO_2_RPN8* (*tetO_2_*, tetracycline-regulatable promoter) and *tetO_2_RPN11*, were purchased from Openbiosystems. Gene silencing was induced by addition of 20 μg/ml tetracycline to growth media at *D* (600 nm)=0.5 and cells growth for indicated duration.

### Glycerol gradient analysis

WCE containing 2–4 mg soluble protein was stacked on a 12 ml of 10%–40% glycerol gradient and ultracentrifuged at 100000 ***g*** for 20 h. One millilitre fractions were collected.

### Proteasome and recombinant protein complex purification

26S or lidless base-CP proteasomes were purified from WT or *rpn11–m1* yeast as described previously [16,69,70]. For expression of recombinant proteasome subunits in Rossetta cells (BL-21 with tRNA), genes were cloned into pETDuet (Novagen). In all subunit combinations, Rpn11 was tagged by His6 at N-terminus for affinity purification by Ni–NTA (HisTrapHQ 5 ml; GE open-biosystems; buffer containing 50 mM Tris, pH 7.4, 5% glycerol, NaCl 100 mM, imidazole 5–280 Mm) followed by size exclusive column (S400 120 ml; GE openbiosystems, 50 mM Tris, pH 7.4, 5% glycerol, NaCl 100 mM).

### Proteasome resolution

WCE from yeast cells was resolved by 4% non-denaturing-PAGE [55]. The peptidase activity based on LLVY–AMC tracing was the marker for cutting the gel slices. The native gel slices were modified with 100 mM iodoacetamide in 10 mM ammonium bicarbonate (at room temperature for 30 min) and trypsinized in 10 mM ammonium bicarbonate containing trypsin [modified trypsin (Promega)] at a 1:50 enzyme-to-substrate ratio, overnight at 37°C.

### MS analysis

The resulting tryptic peptides were resolved by reverse-phase chromatography on 0.075×200 mm fused silica capillaries (J&W) packed with Reprosil reversed phase material (Dr Maisch GmbH, Germany). The peptides were eluted with linear 65 min gradients of 5%–45% and 15 min at 95% acetonitrile with 0.1% formic acid in water at flow rates of 0.25 μl/min. MS was performed by an ion-trap mass spectrometer (Orbitrap, Thermo) in a positive mode using repetitively full MS scan followed by collision induces dissociation (CID) of the seven most dominant ions selected from the first MS scan.

### Database search

The MS data were analysed using the Trans Proteomic Pipeline (TPP) Version 4.3 [72]. TPP-processed centroid fragment peak lists in mzXML format were searched against *Saccharomyces cerevisiae* translations of all systematically named ORFs (open reading frames; http://www.yeastgenome.org/). The proteins were supplemented with their corresponding decoy sequences (as described in http://www.matrixscience.com/help/decoy_help.html). The database searches were performed using X! Tandem with *k*-score plugin through the TPP. Search parameters include: trypsin cleavage specificity with two missed cleavage, cysteine carbamidomethyl as fixed modification, methionine oxidation and protein N-terminal acetylation as variable modifications, peptide tolerance and MS/MS [35].

### Preparation of Ub dimers

Fully natural Ub dimers linked via Lys^6^, Lys^11^, Lys^27^, Lys^29^ and Lys^33^ were synthesized from recombinant Ub monomers using a non-enzymatic chain assembly method according to published protocol [73]. Monomeric Ub mutants, E2 conjugating enzymes and human E1 were purified from recombinant sources as described [74,75]. Enzymatically synthesized Lys^11^-, Lys^48^- and Lys^63^-linked Ub dimers were assembled by combining a proximally blocked Ub mutant (UbD77 or 6His-Ub) in combination with a distally blocked lysine to arginine Ub variant as published [74,76]. Lys^11^-linked dimers were obtained from a reaction containing 10 mg of each 6His-Ub and UbK11R/K63R, 500 nM UBE1, 30 μM Ube2s, 5 mM TCEP and 15 mM ATP in a volume of 2 ml with a 50 mM Tris, pH 8.0, buffer incubated at 30°C for 20 h. Lys^48^-linked dimers were obtained in a similar reaction with E2–25K as the sole E2 and UbD77 and UbK48R/K63R monomers. In a similar fashion, reactions to generate Lys^63^-linked dimers contained Ubc13 (ubiquitin-conjugating enzyme 13)–Uev1a (Ubc variant 1a) and UbD77 and UbK48R/K63R monomers. Following the completion of each reaction, 10 ml of cation buffer A (50 mM ammonium acetate, pH 4.5) was added, the solution was centrifuged at 14000 ***g*** for 10 min to remove precipitated E1 and E2 enzymes and the supernatant was injected on to a 5 ml cation-exchange (SP GE Life Sciences) column at 0.2 ml/min. The polyUb species were eluted with cation buffer B (50 mM ammonium acetate, 1 M NaCl, pH 4.5), exchanged into PBS buffer, pH 7.4, and concentrated to a final volume of 1 ml. Monomeric and dimeric Ub species were separated on a Superdex 75 size exclusion column (GE Life Sciences) in PBS buffer, pH 7.4, with a flow rate of 0.35 ml/min. Fractions containing pure dimers were detected using SDS/PAGE.

## RESULTS

### Suppression of RPN8 or RPN11 disrupts lid assembly

**Figure 1:**
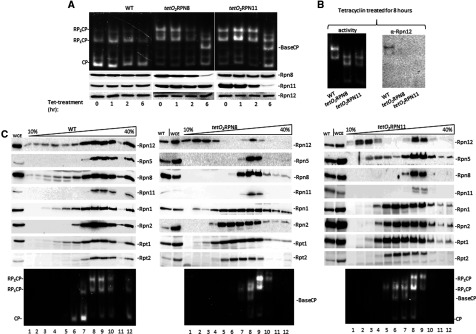
Silencing *RPN8* or *RPN11* suppresses lid biogenesis (**A**) *RPN8* and *RPN11* under control of the tetO_2_ were silenced by addition of 20 μg/ml tetracycline to growth media. At indicated time points, WCE was resolved by 4% non-denaturing (native)-PAGE (top) and 12% SDS/PAGE (bottom). Proteasome activity was traced by in-gel peptidase activity. Effect of tetracycline gene repression on cellular levels of proteasome lid subunits was monitored by immunoblotting specific antibodies as indicated. Majority of proteasomes in untreated cells migrated as doubly and singly capped 26S proteasomes (RP_2_CP, RP_1_CP respectively) and free 20S CP. After 6-h-treatment, faster migration species becomes apparent (top panel), concomitant with ablation of the target gene product in WCE (Rpn8 or Rpn11 accordingly; bottom panels). Composition of this new species was confirmed as base-CP lacking all lid subunits (Table 3). (**B**) Rpn12 ejected from *tetO_2_RPN8* and *tetO_2_RPN11* proteasomes. Eight hours after tetracycline treatment, WCE was resolved by native-PAGE and immunoblotted for presence of Rpn12 in proteolytically-active species. (**C**) Six hours following tetracycline treatment, heterogeneous proteasome species were resolved by fractionating native WCE (as in panel **A**) through a 10%–40% glycerol gradient. Each fraction was assayed for proteasome subunits (top panels) or proteolytic activity (bottom).

Proteasome lid subunits are essential proteins for budding yeast viability; knocking-down any lid subunit (other than Rpn15/Sem1) in this organism results in lethality [5]. We employed inducible gene silencing to study proteasome integrity upon conditional loss of individual lid MPN subunits. Expression of *RPN8* and *RPN11* was placed under control of the repressible *tetO_2_* promoter, in order to repress their transcription upon addition of tetracycline directly to media [77]. Six to eight hours after tetracycline treatment, the levels of Rpn8 or Rpn11 in *tetO_2_RPN8* or *tetO_2_RPN11* strains respectively, decreased well below stoichiometry relative to other proteasome subunits (Figure 1A). Exposure to tetracycline had no effect on the expression of *RPN12*, a result that served to evaluate expression levels of non-engineered proteasome subunits (Figure 1A). Depletion of Rpn8 or Rpn11 in cells reduced levels of doubly- and singly-capped 26S holoenzymes (RP_2_CP, RP_1_CP). Concomitant appearance of faster migrating species was apparent (Figure 1A). Stalled proteasome assembly was indicated by Rpn12 failing to incorporate into newly synthesized proteasomes, limited in either Rpn8 or Rpn11 (Figure 1B). Migration of these proteasomes and composition determined by MS/MS (Table 3) resembled that of ‘lidless’ proteasomes previously identified upon deletion of *RPN10* [16]. Repression of *RPN8* or *RPN11* expression for longer periods (~24 h) resulted in depletion of their gene products to below detection levels, growth arrest and eventual abrogation of proteasome complexes (Supplementary Figures S1 and S2). Interestingly, residual 20S CP remained in these arrested cells (Supplementary Figures S1 and S2). Persistent 20S CP in cells under a variety of stress conditions has been reported [53,55].

**Table 3:** Subunits composition of Proteasome complexes from WT vs lid mutants. Proteasome complexes from WT or various mutants were traced in gels by peptidase activity and subunit composition determined by trypsinization and MS/MS analysis. The number of unique peptides of each identified proteasome subunit is listed. Complexes are marked in Figures 2 and 3

Strain:	WT	tetO_2_–RPN8	tetO_2_–RPN11	rpn11–m1	rpn8–1				rpn8–1	
Complex:	Proteasome	Base-CP	Base-CP	Proteasome (α)	a	b	c	d	Proteasome *in vivo*	Proteasome *ex vivo*
Rpt1	9	13	9	17	8	12	21	7	20	22
Rpt2	4	6	4	12	3	6	9	5	10	17
Rpt3	4	3	2	10	8	14	12	6	13	17
Rpt4	3	7	3	10	8	11	16	4	17	18
Rpt5	5	8	2	20	6	11	17	3	18	23
Rpt6	4	6	3	15	4	9	13	7	17	24
Rpn1	11	11	7	19	14	22	28	4	31	27
Rpn2	25	15	9	18	16	25	29	9	36	49
Rpn13	2	2	1	–	1	3	5	–	2	3
Ubp6	2	3	1	–	6	9	4	1	11	19
Rpn10	2	–	–	–	2	–	17	–	–	1
Rpn3	12	–	–	–	5	7	13	–	8	8
Rpn5	4	–	–	2	4	6	11	–	8	8
Rpn6	6	–	–	9	6	4	10	–	8	5
Rpn7	3	–	–	–	4	4	9	–	4	4
Rpn8	6	–	–	2	2	3	6	–	4	3
Rpn9	12	–	–	7	9	6	11	–	13	4
Rpn11	5	–	–	2	6	5	11	–	5	10
Rpn12	2	–	–	–	4	2	9	–	1	1

Distribution of subunits between proteasome bound and unbound states was probed by centrifuging WCE through a glycerol density gradient. Most proteasome subunits migrated primarily in high *M*_r_ fractions consistent with 26S proteasome holoenzymes (Figure 1C; left). Subunits of the proteasome base sub-complex, represented in this case by Rpn1, Rpn2, Rpt1 and Rpt2, were particularly synchronized with proteolytic activity (lower panels), indicating that most were in complex with 20S CP. Representative lid subunits, Rpn5, 8, 11 and 12, were also enriched in fractions containing proteasome holoenzymes, but trace amounts were found in lower *M*_r_ fractions, suggesting that a portion of lid subunits does not associate with 26S holoenzymes. Six hours after suppression of *RPN8* or *RPN11* expression, a proteasome specie containing all base subunits but lacking lid components was detectable (Figure 1C). Migration patterns of the majority of lid subunits (including the residual Rpn8 and Rpn11 subunits that remained after suppression of their expression) were synchronized with migration of 26S proteasome holoenzymes. A notable exception was Rpn12, a significant portion of which was found detached from proteasomes in extracts from wild-type (WT) and even more so after suppression of *RPN8* or *RPN11* expression (Figure 1C). That Rpn12 is one of the lid assembly intermediates [60] may explain why this subunit is able to remain stable and soluble in a proteasome-unbound state, in contrast with most other proteasome subunits that did not accumulate unassociated from proteasomes. Another subunit that was detected in fractions without peptidase activity characteristic of 20S CP was Rpn5 (Figure 1C), in line with its dual association with COP9 signalosome (CSN) and proteasome complexes [35].

### Partially assembled lid identified in proteasome species from rpn11–m1

**Figure 2:**
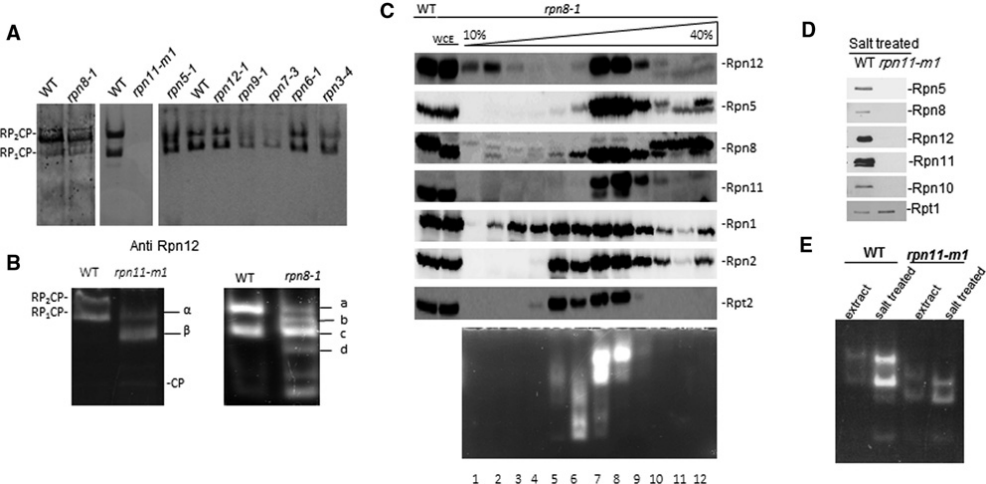
Participation of Rpn8 and Rpn11 C-termini in proteasome stability (**A**) WCE from mutants encoding proteasome lid subunits were resolved by non-denaturing-PAGE and immunoblotted with anti-Rpn12. (**B**) WCE from *rpn8–1* and *rpn11–m1* were also assayed for proteasome activity by ‘*in-gel peptidase activity*’. Subunits composition of indicated species is summarized in Table 3. (**C**) WCE from *rpn8–1* was fractionated through a 10%–40% glycerol gradient and each fraction evaluated for proteasome subunits (top panels) or proteolytic activity (bottom). (**D**) Proteasomes from WT and *rpn11–m1* were exposed to 300 mM NaCl and re-purified. Composition was estimated by immunoblotting for proteasome subunits representing each of the 19S-RP sub-complexes. (**E**) Migration of proteasomes from WT and *rpn11–m1* in native gel before and after exposure to 300 mM NaCl.

Complete loss of lid from proteasome complexes in absence of Rpn8 or Rpn11 was unable to sustain growth over time (Supplementary Figures S1 and S2). In order to obtain information on their role in proteasome lid stability, we compared the outcomes of C-terminal truncations in each of the eight PCI and MPN domain lid subunits grown at their permissive temperature (Figure 2A). Proteasomes isolated from *rpn11–m1* were unique in lacking any detectable incorporated Rpn12 (Figure 2A). Proteasomes from this strain grow at the permissive temperature were proteolytically active but migrated faster than 26S holoenzymes suggesting a more substantial defect than merely loss of labile Rpn12 (Figure 2B). Comparative MS/MS analysis of proteasome complexes from *rpn11–m1* and WT detect substantial loss of three lid subunits (Rpn3, 7 and 12) as well as the non-essential (peripheral) base subunits (Rpn10, Rpn13 and Ubp6) (Table 3). Base-CP, without any detectable lid subunits, typical of proteasomes purified in absence of Rpn10 [16], was also observed (Figure 2B left and result not shown). *rpn8–1* partially emulated this feature; a proteasome specie lacking Rpn10 as well as complete lidless proteasomes alongside singly- and doubly-capped 26S proteasome holoenzymes (Figure 2B; Table 3). Glycerol gradient fractionation confirmed base-CP proteasome species lacking lid subunits in this mutant (Figure 2C).

Absence of Rpn10 has been shown previously to render proteasome holoenzymes fragile and particularly sensitive to salt leading to rapid dissociation of entire lid after exposure to ~300 mM NaCl [16]. Proteasomes from *rpn11–m1* were exposed to low 300 mM NaCl in buffer and re-isolated. No lid subunits remained stably associated with base-CP complexes, in contrast with 26S proteasomes from WT that were resilient to this treatment (Figure 2D). Following salt treatment, proteasomes from *rpn11–m1* migrated slightly faster by non-denaturing gels (Figure 2E), further supporting change in subunit composition (loss of residual lid subunits). Likewise, MS/MS did not pick up peptides derived from lid subunits in these samples (3). Consequentially, we wish to comment that proteasome subunit composition should not be concluded solely from migration patterns in non-denaturing gels. Although the standard protocols for native gels are powerful in distinguishing 20S CP from singly- and doubly-capped 26S holoenzymes, changes within 19S and lid seem much harder to resolve, even when involving multiple subunits.

### C-terminal helix of Rpn11 recruits labile proteasome lid subunits

**Figure 3:**
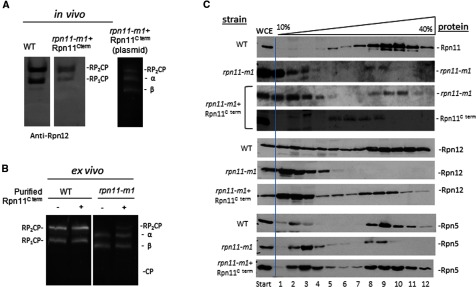
The C-terminus of Rpn11 is capable of recruiting missing module 2 subunits (**A**) The C-terminus fragment of Rpn11 was expressed as a separate gene product in *rpn11–m1* background; proteasome species were compared with WT by in-gel peptidase activity (right) followed by immunoblotting by anti-Rpn12 (left). (**B**) WCE of *rpn11–m1* (or WT) was incubated for 30 min with or without a recombinant polypeptide corresponding to the C-terminus of Rpn11; proteasomes were then visualized by in-gel peptidase activity. (**C**) WCE of *WT*, *rpn11–m1* and *rpn11–m1* expressing the C-terminal fragment were resolved by glycerol gradient; all fractions were immunoblotted by anti-Rpn11 to monitor distribution of Rpn11 or its fragments.

Three lid subunits, Rpn3, Rpn7 and Rpn12, were not detected in proteasomes isolated from a mutant lacking the last 31 amino acids of Rpn11 (Figure 2; Table 3). Nevertheless, absence of this Rpn11 tail did not abrogate the ability of module 1 subunits in the lid to bind base-CP and give rise to a previously undocumented incomplete proteasome complex. Co-expression of both Rpn11 fragments restored typical proteasome configuration even though the two domains of Rpn11 were not physically bound to each other (Figure 3A) [78,79]. Moreover, addition of a recombinant polypeptide identical in sequence to the C-terminal segment of Rpn11 *rpn11–m1* extracts was sufficient to generate 26S proteasome holoenzymes, apparently by recruiting free module subunits (Figure 3B). In either case, complete 26S holoenzymes composition was confirmed by MS/MS (Table 3). These experiments demonstrate distinct structural roles for the MPN and C-terminal domains of Rpn11. Whereas the MPN domain appears sufficient to recruit co-ordinate Rpn11 into module 1 and incorporation into proteasomes, the C-terminal segment emerges as critical for retaining module 2 subunits in 19S RP of the 26S holoenzymes. Notably, both fragments of Rpn11 were present in tandem in reconstituted holoenzymes, even if not physically attached (Figure 3C). The labile subunit Rpn12 was re-incorporated into proteasomes merely by presence of the C-terminal fragment of Rpn11 (Figure 3C), further supporting a role for Rpn11 in linking modules 1 and 2.

### The minimal lid composition competent to bind base-CP

**Figure 4:**
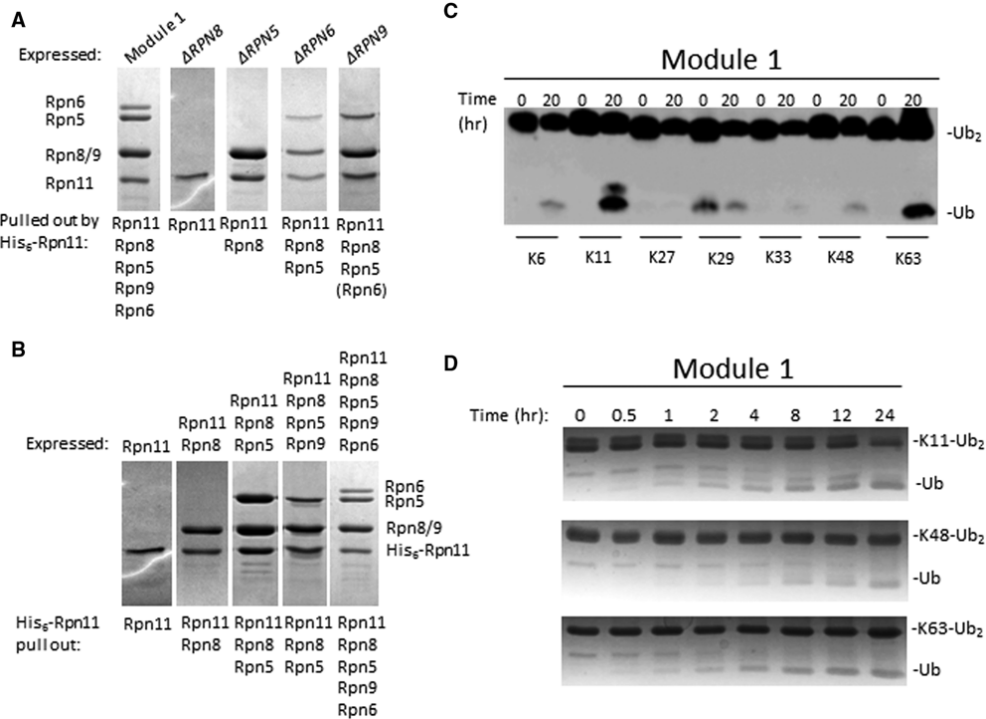
Reconstitution of proteasome lid mini-complexes revolving around Rpn11 (**A**) Recombinant His_6_–Rpn11 was co-expressed in *E*. *coli* alongside all lid module 1 subunits (Rpn5, Rpn6, Rpn8 or Rpn9 respectively) or in various combinations lacking one of the subunits from lid module 1 (Rpn5, Rpn6, Rpn8 and Rpn9). His_6_-tagged Rpn11 and associated proteins were tandem affinity purified and evaluated for composition (identify of all protein bands were confirmed by MS/MS). (**B**) Recombinant His_6_–Rpn11 was expressed in *E*. *coli*, in various combinations with other subunits from lid module 1 (Rpn5, Rpn6, Rpn8 and Rpn9). His_6_-tagged Rpn11 and associated proteins were tandem affinity purified and evaluated for composition (identify of all protein bands were confirmed by MS/MS). (**C**) Recombinant module 1 is an active DUBs. Fully natural Ub dimers linked via Lys^6^, Lys^11^, Lys^27^, Lys^29^, Lys^33^, Lys^48^ or Lys^63^ were synthesized from recombinant Ub monomers. Ten micromolar of each Ub dimer was incubated with 1 μM of purified recombinant module 1. (**D**) Ten micromolar of enzymatically synthesized Lys^11^–Ub_2_, Lys^48^–Ub_2_ or Lys^63^–Ub_2_ were incubated with 1 μM of the indicated enzyme and visualized by Coomassie stained SDS/PAGE.

By characterization of proteasome species in mutants, we have learned that it is possible for proteasome complexes to contain only a portion of lid subunits. Module1–base-CP proteasomes could reflect transiently-associated module 2 that easily detaches during isolation procedures or an assembly intermediate that has difficulty to recruit module 2 due to the mutation in Rpn11. In order to demonstrate whether module 1 can self-assemble and bind directly to base-CP, we generated a recombinant module 1 complex. Tagged Rpn11 was co-expressed in various combinations with other module 1 subunits in *Escherichia coli* and isolated by Ni–NTA affinity purification followed by size exclusion chromatography. Without Rpn8, no complexes of Rpn11 with other lid subunits were detected (Figure 4A). Heterodimerization of Rpn8 and Rpn11 yielded stable dimers independent of any other factors (Figures 4A and 4B). Indeed, dimerization is probably mediated by their MPN domains [13,61]. In absence of Rpn5, this Rpn8–Rpn11 dimer was unable to recruit other lid subunits. Next, a stable Rpn5–Rpn8–Rpn11 trimer was independent of Rpn6 or Rpn9 (Figure 4A). A sequential order of subunit addition during module 1 assembly was confirmed by a stepwise expression of subunits (Figure 4B). Co-expression of all five subunits self-assembled into a stable module 1 (Figures 4A and 4B). The resulting module 1 was validated for its inherent deubiquitination activity. Homogenously-linked dimeric Ub linked through each of the seven possible lysines, was incubated with module 1 and products separated by SDS/PAGE. Monoubiquitin was generated primarily from Lys^11^–Ub2 and for Lys^63^–Ub2, reported to be the preferred linkages of Rpn11 [13], and to a lesser extent from Lys^48^, Lys^6^ and even Lys^33^ Ub2 (Figure 4C). A reaction time course demonstrated deubiquitination activity by module 1 for Lys^11^, Lys^63^ and Lys^48^ linked substrates (Figure 4D).

**Figure 5:**
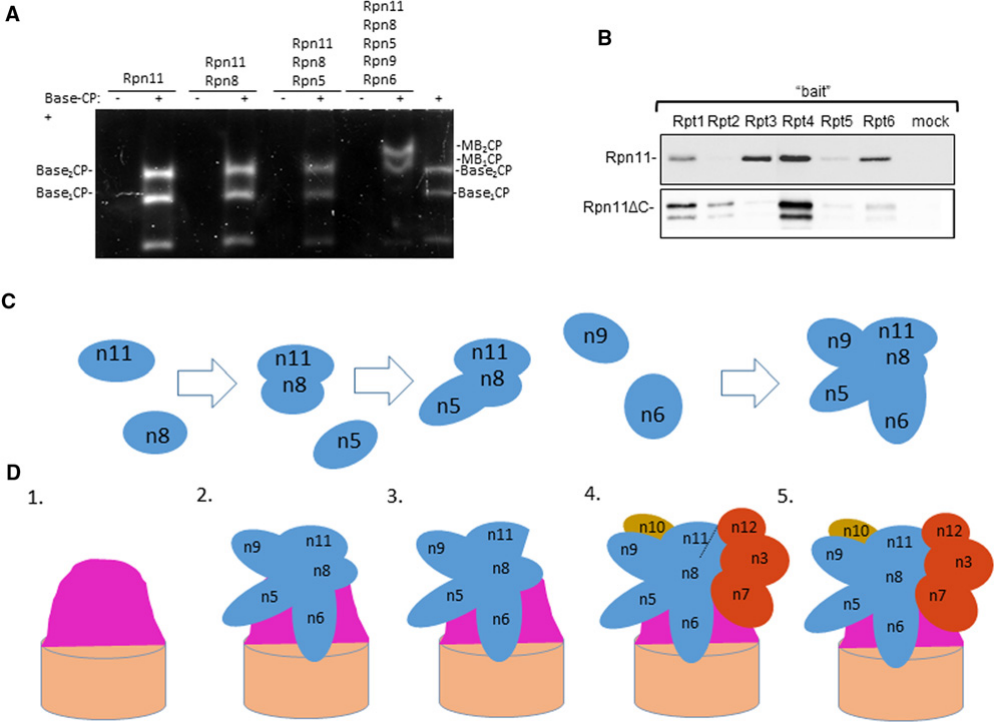
Base-CP can serve as a platform for stepwise lid formation (**A**) Stably purified lid intermediates from Figure 4(**B**) were incubated with purified base-CP (equivalent to lidless proteasome) and resulting association monitored by proteolytic assay on native gel. (**B**) Specific interactions of Rpn11 with RPT ATPases depends on C-terminal fragment. Each RPT ATPase was purified and immobilized on CH-sepharose beads and incubated with either full-length Rpn11 or Rpn11ΔC. Beads with immobilized BSA were used as a negative control (mock). Bound proteins were separated on SDS/PAGE and immunoblotted with anti-Rpn11. (**C**) Assembly pathway of lid module 1. Relative orientation of subunits is based on PDB 4CR2. (**D**) Module 1 can serve as a *de facto* lid core. A summary of proteasome species identified in the present study, from left to right: 1. base-CP, 2. module1–base-CP, 3. incomplete 26S identified in *rpn11–m1* containing a lid core, 4. proteasomes from *rpn11–m1* upon addback of Rpn11 C-terminal fragment and 5. 26S proteasome holoenzymes. Relative orientation of lid subunits running along the side of the base (illustrated as a pink mound) is based on PDB 4CR2. Schematic depiction of main sub-complexes and key lid subunits as follows: brown cylinder, 20S CP; pink mound, base; blue, lid core (module 1) subunits; red, labile (module 2) lid subunits; violet, Rpn10 bridging lid and base.

**Figure 6:**
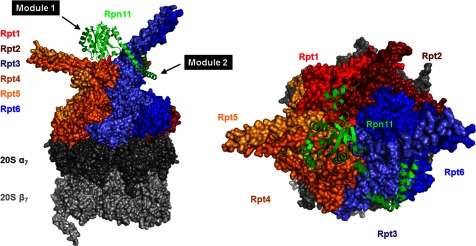
Prying open the two domains of Rpn11 in the 26S proteasome Position of Rpn11 (green) in 26S holoenzymes from EM model (PDB-4CR2) supports distinct interactions of MPN domain and C-terminal region. Lid PCI domains and Ub-processing factors in the base (Rpn1, Rpn2, Rpn10 and Rpn13) were rendered invisible in order to highlight the relative position of Rpn11 (green) to the ring of RPT ATPases. Rpt1 (red), Rpt2 (dark red), Rpt3 (light blue), Rpt4 (dark orange), Rpt5 (orange) and Rpt6 (blue). The hexameric RPT ring locks on to the 20S CP made up of the distal α-heptamer (dark grey) and the proteolytic β-heptamer (light grey). Note the pairing of RPTs via N-terminal coiled-coils: Rpt1–Rpt2, Rpt4–Rpt5 and Rpt3–Rpt6. Rpn11 catalytic MPN domain is situated directly above the centre of this hexameric RPT ring with its C-terminal segment pried away wrapping around the Rpt3–Rpt6 coiled-coil. The unique orientation of Rpn11 enables it to bridge between lid module 1 and module 2: remainder of module 1 subunits (Rpn5, Rpn6, Rpn8 and Rpn9) co-ordinate around the MPN domain of Rpn11, whereas the α-helix at its C-terminus participates in a helix bundle with C-termini of its paralogue Rpn8 and module 2 subunits (Rpn3, Rpn7 and Rpn12).

Stable mini-complexes of module 1 were tested whether they were able to integrate into proteasomes by association with pre-assembled base-CP, purified as published [16]. The only complex competent to attach to base-CP was module 1 that included Rpn6 (Figure 5A). In this manner, it was possible to reconstitute *in vitro*, a module1–base-CP complex, identical in composition to incomplete proteasomes abundant in *rpn11–m1* (Table 3). Recent EM studies position Rpn11 at the centre of proteasome holoenzyme, situated directly above the hexameric ring of ATPases [18,27]. To obtain biochemical insight as to which RPTs Rpn11 is in close contact with, we tested pairwise associations of Rpn11 with each ATPase. We found that Rpt1, Rpt3, Rpt4 and Rpt6 formed stable associations with Rpn11 (Figure 5B). Even lacking its C-terminal residues, Rpn11 was incorporated into proteasomes (Figure 3). Interestingly, this truncated reduced the ability to bind Rpt6 and Rpt3, yet retained stable association with Rpt1 and Rpt4 (Figure 5B). We conclude that within the proteasome holoenzyme, the C-terminal region of Rpn11 interacts with distinct partners from those that associate with its MPN domain. These interactions orient module 2 within the 19S RP (Figure 6).

## DISCUSSION

Proteasomes are strictly required for viability of all eukaryotic cells. Nevertheless, fragile complexes or proteasomes lacking certain subunits have been documented in mutants or under stress conditions. For this reason, mutants have been instrumental in dissecting assembly pathways, mapping nearest neighbour interactions and determining complex stability [3,34,42,43,49,51,59,66,67,78,80–84]. Similarly, by knocking down either of the MPN-subunits, Rpn11 or Rpn8, we have demonstrated that base was still assembled in cells associated to 20S CP. Heterodimerization of Rpn8–Rpn11 appeared to be a key step for initiation of lid assembly, followed by addition of Rpn5, with Rpn9 and Rpn6 coming in last to form module 1 (Figure 5C). The resulting five-subunit complex corresponds to lid assembly intermediate module 1, which we now demonstrate is competent to bind base-CP (Figure 5D). Once incorporated into proteasomes, module 1 serves in effect as the ‘lid core’ sustaining a proteasome species prevalent in certain mutants and easily reconstituted from isolated components *in vitro* (Figure 5D). The current study provides evidence that module 2 subunits are present in cell extract and are able to re-attach to proteasome complexes. However, we have no evidence, thus far, whether module 2 exists as an independent stable complex when detached from module 1. The Rpn11 C-terminus was demonstrated as a critical factor in stabilizing incorporation of module 2 subunits on to 26S holoenzymes. The positon of Rpn11 obtained from high-resolution EM models highlights the bipartite nature of its structure (Figure 6), its centrally located MPN domain co-ordinates module 1, whereas the C-terminus wraps around Rpt3–Rpt6 coiled-coil extension positioned to anchor module 2 in the 19S RP. Subunit arrangement within the lid and its overall architecture is remarkably similar to that of the CSN complex [36], yet in contrast to Rpn11, Csn5, the active metallo-protease and direct paralogue of Rpn11, is labile and can be the last to incorporate or first to detach [85]. Differences in assembly and stability of these two complexes may control of their respective enzymatic activities; whereas CSN is maintained as an inactive protease until bound to its Cullin substrate [85–87], lid and module 1 are active relative to free Rpn11 (Figure 4) [10,13].

Isolated Rpn11 is latent, yet can be partially activated either when incorporated into 26S proteasome holoenzymes or by truncation of its C-terminal sequence [13]. The current study provides a possible explanation; repression of Rpn11 catalytic activity by its C-terminal tail may be alleviated by a conformational change that distances the C-terminus from the globular MPN domain as occurs in the proteasome (Figure 6). Seeing as Rpn11 in module 1 displays elevated rates of DUB activity compared to Rpn11 or Rpn11–Rpn8 heterodimer [13], we propose that conformational changes upon binding of Rpn5 or other neighbours in module 1 (Figures 4 and 5) may be sufficient to partially alleviate repression by the C-terminus of Rpn11. Such an allosteric effect may be a boon to enzymatic studies of Rpn11 properties and facilitate screening of inhibitors. Although the C-terminus of Rpn5 has not been proposed to participate in the tight helix bundle composed of the extreme C-termini of most lid subunits [61], our biochemical data establish a stable association between Rpn5 the Rpn8–Rpn11 heterodimer. This association is likely to be through its C-terminus [34,35]. Furthermore, this trimeric complex is a prerequisite for formation of module 1.

Multi-subunit complexes, such as the proteasome, may enlist multiple assembly pathways to guarantee robust production. In the present study, module 1 was assembled independently and was found to be competent to bind base-CP, however in the crowded milieu of the cytosol, other assembly pathways may exist in parallel. For instance, complete assembly of lid [34], stepwise assembly of lid on base initiated by one of the subunits with highest affinity for base (such as Rpn6 [88] or Rpn11 [51]) or complete preassembly of the 19S RP before attachment to 20S [17,60] are alternative pathways that may each lead to 26S proteasome assembly. Once assembled, 19S RP and 20S CP may dissociate in cells or in biochemical preparations [48,52–54], although disassembly and assembly need not to follow an identical itinerary. The equilibrium of 19S RP with 20S CP can be perturbed by external stress conditions, senescence, neurodegeneration, aging or influenced through mutation of subunits [16,34,51,55,66,67,89–92].

Characterization of the module1–base-CP intermediate provides new information for the importance of Rpn6 in determining proteasome stability [93,94]. Rpn6 is an elongated super-helical subunit that physically links all three sub-complexes: lid, base and CP (Figure 5). Beyond participating in the helix bundle tying lid subunits together, Rpn6 also simultaneously interacts with Rpt6 and α2 [66,88]. This property explains the decisive role that Rpn6 has in partitioning between 19S assembly pathways, depending on availability of partners and relative strength of interactions. Similarly, Rpn11 also bridges several RPTs, module 1 and module 2 subunits. Module 1 apparently revolves around the MPN domain heterodimer of Rpn8 and Rpn11. The current study also illuminates a critical role for the C-terminal residues of Rpn11 in docking of module 2 components (Figures 5D and 6). It is notable that all module 2 subunits participate along with Rpn11 in the helix bundle through their respective C-termini. This may explain why module 2 subunits were not found in proteasomes studied from the *rpn11–m1* mutant (Figures 2–3). Identifying a new proteasome species in an *rpn11* mutant is an insightful development towards charting alternative routes for biogenesis of 26S proteasome holoenzymes and for defining functional units within this intricate machine.

## Online data

Supplementary data

## References

[1] Schwanhausser B., Busse D., Li N., Dittmar G., Schuchhardt J., Wolf J., Chen W., Selbach M. (2013). Corrigendum: global quantification of mammalian gene expression control. Nature.

[2] Komander D. (2009). The emerging complexity of protein ubiquitination. Biochem. Soc. Trans..

[3] Finley D. (2009). Recognition and processing of ubiquitin-protein conjugates by the proteasome. Annu. Rev. Biochem..

[4] Glickman M.H., Ciechanover A. (2002). The ubiquitin-proteasome proteolytic pathway: destruction for the sake of construction. Physiol. Rev..

[5] Glickman M.H., Rubin D.M., Fried V.A., Finley D. (1998). The regulatory particle of the *Saccharomyces cerevisiae* proteasome. Mol. Cell. Biol..

[6] Tanaka K., Mizushima T., Saeki Y. (2012). The proteasome: molecular machinery and pathophysiological roles. Biol. Chem..

[7] Inobe T., Matouschek A. (2008). Protein targeting to ATP-dependent proteases. Curr. Opin. Struct. Biol..

[8] Peth A., Uchiki T., Goldberg A.L. (2010). ATP-dependent steps in the binding of ubiquitin conjugates to the 26S proteasome that commit to degradation. Mol. Cell.

[9] Peth A., Nathan J.A., Goldberg A.L. (2013). The ATP costs and time required to degrade ubiquitinated proteins by the 26 S proteasome. J. Biol. Chem..

[10] Guterman A., Glickman M.H. (2004). Complementary roles for Rpn11 and Ubp6 in deubiquitination and proteolysis by the proteasome. J. Biol. Chem..

[11] Glickman M.H., Adir N. (2004). The proteasome and the delicate balance between destruction and rescue. PLoS Biol..

[12] Inobe T., Matouschek A. (2014). Paradigms of protein degradation by the proteasome. Curr. Opin. Struct. Biol..

[13] Mansour W., Nakasone M.A., von Delbrueck M., Yu Z., Krutauz D., Reis N., Kleifeld O., Sommer T., Fushman D., Glickman M.H. (2014). Disassembly of Lys11- and mixed-linkage polyubiquitin conjugates provide insights into function of proteasomal deubiquitinases Rpn11 and Ubp6. J. Biol. Chem..

[14] Guterman A., Glickman M.H. (2004). Deubiquitinating enzymes are IN/(trinsic to proteasome function). Curr. Protein Pept. Sci..

[15] Lee M.J., Lee B.H., Hanna J., King R.W., Finley D. (2011). Trimming of ubiquitin chains by proteasome-associated deubiquitinating enzymes. Mol. Cell. Proteomics.

[16] Glickman M.H., Rubin D.M., Coux O., Wefes I., Pfeifer G., Cjeka Z., Baumeister W., Fried V.A., Finley D. (1998). A subcomplex of the proteasome regulatory particle required for ubiquitin-conjugate degradation and related to the COP9-signalosome and eIF3. Cell.

[17] Lander G.C., Estrin E., Matyskiela M.E., Bashore C., Nogales E., Martin A. (2012). Complete subunit architecture of the proteasome regulatory particle. Nature.

[18] Lasker K., Forster F., Bohn S., Walzthoeni T., Villa E., Unverdorben P., Beck F., Aebersold R., Sali A., Baumeister W. (2012). Molecular architecture of the 26S proteasome holocomplex determined by an integrative approach. Proc. Natl. Acad. Sci. U.S.A..

[19] Rosenzweig R., Bronner V., Zhang D., Fushman D., Glickman M.H. (2012). Rpn1 and Rpn2 coordinate ubiquitin processing factors at proteasome. J. Biol. Chem..

[20] Maytal-Kivity V., Reis N., Hofmann K., Glickman M.H. (2002). MPN+, a putative catalytic motif found in a subset of MPN domain proteins from eukaryotes and prokaryotes, is critical for Rpn11 function. BMC Biochem..

[21] Worden E.J., Padovani C., Martin A. (2014). Structure of the Rpn11-Rpn8 dimer reveals mechanisms of substrate deubiquitination during proteasomal degradation. Nat. Struct. Mol. Biol..

[22] Ambroggio X.I., Rees D.C., Deshaies R.J. (2004). JAMM: a metalloprotease-like zinc site in the proteasome and signalosome. PLoS Biol..

[23] Sanches M., Alves B.S., Zanchin N.I., Guimaraes B.G. (2007). The crystal structure of the human Mov34 MPN domain reveals a metal-free dimer. J. Mol. Biol..

[24] Pathare G.R., Nagy I., Sledz P., Anderson D.J., Zhou H.J., Pardon E., Steyaert J., Forster F., Bracher A., Baumeister W. (2014). Crystal structure of the proteasomal deubiquitylation module Rpn8-Rpn11. Proc. Natl. Acad. Sci. U.S.A..

[25] Bhattacharyya S., Yu H., Mim C., Matouschek A. (2014). Regulated protein turnover: snapshots of the proteasome in action. Nat. Rev. Mol. Cell. Biol..

[26] Matyskiela M.E., Lander G.C., Martin A. (2013). Conformational switching of the 26S proteasome enables substrate degradation. Nat. Struct. Mol. Biol..

[27] Unverdorben P., Beck F., Sledz P., Schweitzer A., Pfeifer G., Plitzko J.M., Baumeister W., Forster F. (2014). Deep classification of a large cryo-EM dataset defines the conformational landscape of the 26S proteasome. Proc. Natl. Acad. Sci. U.S.A..

[28] Verma R., Aravind L., Oania R., McDonald W.H., Yates J.R., Koonin E.V., Deshaies R.J. (2002). Role of Rpn11 metalloprotease in deubiquitination and degradation by the 26S proteasome. Science.

[29] Yao T., Cohen R.E. (2002). A cryptic protease couples deubiquitination and degradation by the proteasome. Nature.

[30] da Fonseca P.C., He J., Morris E.P. (2012). Molecular model of the human 26S proteasome. Mol. Cell.

[31] Fu H., Reis N., Lee Y., Glickman M.H., Vierstra R.D. (2001). Subunit interaction maps for the regulatory particle of the 26S proteasome and the COP9 signalosome. EMBO J..

[32] Sharon M., Taverner T., Ambroggio X.I., Deshaies R.J., Robinson C.V. (2006). Structural organization of the 19S proteasome lid: insights from MS of intact complexes. PLoS Biol..

[33] Davy A., Bello P., Thierry-Mieg N., Vaglio P., Hitti J., Doucette-Stamm L., Thierry-Mieg D., Reboul J., Boulton S., Walhout A.J. (2001). A protein-protein interaction map of the *Caenorhabditis elegans* 26S proteasome. EMBO Rep..

[34] Isono E., Nishihara K., Saeki Y., Yashiroda H., Kamata N., Ge L., Ueda T., Kikuchi Y., Tanaka K., Nakano A., Toh-e A. (2007). The assembly pathway of the 19S regulatory particle of the yeast 26S proteasome. Mol. Biol. Cell.

[35] Yu Z., Kleifeld O., Lande-Atir A., Bsoul M., Kleiman M., Krutauz D., Book A., Vierstra R.D., Hofmann K., Reis N. (2011). Dual function of Rpn5 in two PCI complexes, the 26S proteasome and COP9 signalosome. Mol. Biol. Cell.

[36] Pick E., Hofmann K., Glickman M.H. (2009). PCI complexes: beyond the proteasome, CSN, and eIF3 Troika. Mol. Cell.

[37] Hofmann K., Bucher P. (1998). The PCI domain: a common theme in three multi-protein complexes. Trends Biochem. Sci..

[38] Tomko R.J., Hochstrasser M. (2014). The intrinsically disordered Sem1 protein functions as a molecular tether during proteasome lid biogenesis. Mol. Cell.

[39] Fukunaga K., Kudo T., Toh-e A., Tanaka K., Saeki Y. (2010). Dissection of the assembly pathway of the proteasome lid in *Saccharomyces cerevisiae*. Biochem. Biophys. Res. Commun..

[40] Bohn S., Sakata E., Beck F., Pathare G.R., Schnitger J., Nagy I., Baumeister W., Forster F. (2013). Localization of the regulatory particle subunit Sem1 in the 26S proteasome. Biochem. Biophys. Res. Commun..

[41] Chen C., Huang C., Chen S., Liang J., Lin W., Ke G., Zhang H., Wang B., Huang J., Han Z. (2008). Subunit-subunit interactions in the human 26S proteasome. Proteomics.

[42] Rinaldi T., Pick E., Gambadoro A., Zilli S., Maytal-Kivity V., Frontali L., Glickman M.H. (2004). Participation of the proteasomal lid subunit Rpn11 in mitochondrial morphology and function is mapped to a distinct C-terminal domain. Biochem. J..

[43] Rinaldi T., Hofmann L., Gambadoro A., Cossard R., Livnat-Levanon N., Glickman M.H., Frontali L., Delahodde A. (2008). Dissection of the carboxyl-terminal domain of the proteasomal subunit Rpn11 in maintenance of mitochondrial structure and function. Mol. Biol. Cell.

[44] Bedford L., Paine S., Sheppard P.W., Mayer R.J., Roelofs J. (2010). Assembly, structure, and function of the 26S proteasome. Trends Cell Biol..

[45] Matias A.C., Ramos P.C., Dohmen R.J. (2010). Chaperone-assisted assembly of the proteasome core particle. Biochem. Soc. Trans..

[46] Rosenzweig R., Glickman M.H. (2008). Chaperone-driven proteasome assembly. Biochem. Soc. Trans..

[47] Murata S., Yashiroda H., Tanaka K. (2009). Molecular mechanisms of proteasome assembly. Nat. Rev. Mol. Cell. Biol..

[48] Funakoshi M., Tomko R.J., Kobayashi H., Hochstrasser M. (2009). Multiple assembly chaperones govern biogenesis of the proteasome regulatory particle base. Cell.

[49] Park S., Roelofs J., Kim W., Robert J., Schmidt M., Gygi S.P., Finley D. (2009). Hexameric assembly of the proteasomal ATPases is templated through their C termini. Nature.

[50] Tomko R.J., Funakoshi M., Schneider K., Wang J., Hochstrasser M. (2010). Heterohexameric ring arrangement of the eukaryotic proteasomal ATPases: implications for proteasome structure and assembly. Mol. Cell.

[51] Hendil K.B., Kriegenburg F., Tanaka K., Murata S., Lauridsen A.M., Johnsen A.H., Hartmann-Petersen R. (2009). The 20S proteasome as an assembly platform for the 19S regulatory complex. J. Mol. Biol..

[52] Liu C.W., Li X., Thompson D., Wooding K., Chang T.L., Tang Z., Yu H., Thomas P.J., DeMartino G.N. (2006). ATP binding and ATP hydrolysis play distinct roles in the function of 26S proteasome. Mol. Cell..

[53] Bajorek M., Finley D., Glickman M.H. (2003). Proteasome disassembly and downregulation is correlated with viability during stationary phase. Curr. Biol..

[54] Sawada H., Akaishi T., Katsu M., Yokosawa H. (1997). Difference between PA700-like proteasome activator complex and the regulatory complex dissociated from the 26S proteasome implies the involvement of modulating factors in the 26S proteasome assembly. FEBS Lett..

[55] Livnat-Levanon N., Kevei E., Kleifeld O., Krutauz D., Segref A., Rinaldi T., Erpapazoglou Z., Cohen M., Reis N., Hoppe T., Glickman M.H. (2014). Reversible 26S proteasome disassembly upon mitochondrial stress. Cell Rep..

[56] Beckwith R., Estrin E., Worden E.J., Martin A. (2013). Reconstitution of the 26S proteasome reveals functional asymmetries in its AAA+unfoldase. Nat. Struct. Mol. Biol..

[57] Matiuhin Y., Kirkpatrick D.S., Ziv I., Kim W., Dakshinamurthy A., Kleifeld O., Gygi S.P., Reis N., Glickman M.H. (2008). Extraproteasomal Rpn10 restricts access of the polyubiquitin-binding protein Dsk2 to proteasome. Mol. Cell.

[58] Funakoshi M., Li X., Velichutina I., Hochstrasser M., Kobayashi H. (2004). Sem1, the yeast ortholog of a human BRCA2-binding protein, is a component of the proteasome regulatory particle that enhances proteasome stability. J. Cell. Sci..

[59] Sone T., Saeki Y., Toh-e A., Yokosawa H. (2004). Sem1p is a novel subunit of the 26 S proteasome from *Saccharomyces cerevisiae*. J. Biol. Chem..

[60] Tomko R.J., Hochstrasser M. (2011). Incorporation of the Rpn12 subunit couples completion of proteasome regulatory particle lid assembly to lid-base joining. Mol. Cell.

[61] Estrin E., Lopez-Blanco J.R., Chacon P., Martin A. (2013). Formation of an intricate helical bundle dictates the assembly of the 26S proteasome lid. Structure.

[62] Rinaldi T., Bolotin-Fukuhara M., Frontali L. (1995). A *Saccharomyces cerevisiae* gene essential for viability has been conserved in evolution. Gene.

[63] Rinaldi T., Ricci C., Porro D., Bolotin-Fukuhara M., Frontali L. (1998). A mutation in a novel yeast proteasomal gene, RPN11/MPR1, produces a cell cycle arrest, overreplication of nuclear and mitochondrial DNA, and an altered mitochondrial morphology. Mol. Biol. Cell.

[64] Rinaldi T., Ricordy R., Bolotin-Fukuhara M., Frontali L. (2002). Mitochondrial effects of the pleiotropic proteasomal mutation mpr1/rpn11: uncoupling from cell cycle defects in extragenic revertants. Gene.

[65] Bailly E., Reed S.I. (1999). Functional characterization of rpn3 uncovers a distinct 19S proteasomal subunit requirement for ubiquitin-dependent proteolysis of cell cycle regulatory proteins in budding yeast. Mol. Cell. Biol..

[66] Isono E., Saito N., Kamata N., Saeki Y., Toh E.A. (2005). Functional analysis of Rpn6p, a lid component of the 26 S proteasome, using temperature-sensitive rpn6 mutants of the yeast *Saccharomyces cerevisiae*. J. Biol. Chem..

[67] Isono E., Saeki Y., Yokosawa H., Toh-e A. (2004). Rpn7 is required for the structural integrity of the 26 S proteasome of *Saccharomyces cerevisiae*. J. Biol. Chem..

[68] Takeuchi J., Fujimuro M., Yokosawa H., Tanaka K., Toh-e A. (1999). Rpn9 is required for efficient assembly of the yeast 26S proteasome. Mol. Cell. Biol..

[69] Leggett D.S., Glickman M.H., Finley D. (2005). Purification of proteasomes, proteasome subcomplexes, and proteasome-associated proteins from budding yeast. Methods Mol. Biol..

[70] Glickman M., Coux O. (2001). Purification and characterization of proteasomes from *Saccharomyces cerevisiae*. Curr. Protoc. Protein Sci..

[71] Rosenzweig R., Osmulski P.A., Gaczynska M., Glickman M.H. (2008). The central unit within the 19S regulatory particle of the proteasome. Nat. Struct. Mol. Biol..

[72] Keller A., Eng J., Zhang N., Li X.J., Aebersold R. (2005). A uniform proteomics MS/MS analysis platform utilizing open XML file formats. Mol. Syst. Biol..

[73] Castaneda C., Liu J., Chaturvedi A., Nowicka U., Cropp T.A., Fushman D. (2011). Nonenzymatic assembly of natural polyubiquitin chains of any linkage composition and isotopic labeling scheme. J. Am. Chem. Soc..

[74] Nakasone M.A., Livnat-Levanon N., Glickman M.H., Cohen R.E., Fushman D. (2013). Mixed-linkage ubiquitin chains send mixed messages. Structure.

[75] Volk S., Wang M., Pickart C.M. (2005). Chemical and genetic strategies for manipulating polyubiquitin chain structure. Methods Enzymol..

[76] Castaneda C.A., Kashyap T.R., Nakasone M.A., Krueger S., Fushman D. (2013). Unique structural, dynamical, and functional properties of k11-linked polyubiquitin chains. Structure.

[77] Mnaimneh S., Davierwala A.P., Haynes J., Moffat J., Peng W.T., Zhang W., Yang X., Pootoolal J., Chua G., Lopez A. (2004). Exploration of essential gene functions via titratable promoter alleles. Cell.

[78] Chandra A., Chen L., Liang H., Madura K. (2010). Proteasome assembly influences interaction with ubiquitinated proteins and shuttle factors. J. Biol. Chem..

[79] Chandra A., Chen L., Madura K. (2010). Synthetic lethality of rpn11–1 rpn10Delta is linked to altered proteasome assembly and activity. Curr. Genet..

[80] Glickman M.H., Raveh D. (2005). Proteasome plasticity. FEBS Lett..

[81] Schmidt M., Finley D. (2014). Regulation of proteasome activity in health and disease. Biochim. Biophys. Acta.

[82] Joshi K.K., Chen L., Torres N., Tournier V., Madura K. (2011). A proteasome assembly defect in rpn3 mutants is associated with Rpn11 instability and increased sensitivity to stress. J. Mol. Biol..

[83] Rubin D.M., Glickman M.H., Larsen C.N., Dhruvakumar S., Finley D. (1998). Active site mutants in the six regulatory particle ATPases reveal multiple roles for ATP in the proteasome. EMBO J..

[84] Lin Y.L., Sung S.C., Tsai H.L., Yu T.T., Radjacommare R., Usharani R., Fatimababy A.S., Lin H.Y., Wang Y.Y., Fu H. (2011). The defective proteasome but not substrate recognition function is responsible for the null phenotypes of the *Arabidopsis* proteasome subunit RPN10. Plant Cell..

[85] Lingaraju G.M., Bunker R.D., Cavadini S., Hess D., Hassiepen U., Renatus M., Fischer E.S., Thoma N.H. (2014). Crystal structure of the human COP9 signalosome. Nature.

[86] Birol M., Enchev R.I., Padilla A., Stengel F., Aebersold R., Betzi S., Yang Y., Hoh F., Peter M., Dumas C., Echalier A. (2014). Structural and biochemical characterization of the cop9 signalosome CSN5/CSN6 heterodimer. PLoS One.

[87] Echalier A., Pan Y., Birol M., Tavernier N., Pintard L., Hoh F., Ebel C., Galophe N., Claret F.X., Dumas C. (2013). Insights into the regulation of the human COP9 signalosome catalytic subunit, CSN5/Jab1. Proc. Natl. Acad. Sci. U.S.A..

[88] Pathare G.R., Nagy I., Bohn S., Unverdorben P., Hubert A., Korner R., Nickell S., Lasker K., Sali A., Tamura T. (2012). The proteasomal subunit Rpn6 is a molecular clamp holding the core and regulatory subcomplexes together. Proc. Natl. Acad. Sci. U.S.A..

[89] Byrne A., McLaren R.P., Mason P., Chai L., Dufault M.R., Huang Y., Liang B., Gans J.D., Zhang M., Carter K. (2010). Knockdown of human deubiquitinase PSMD14 induces cell cycle arrest and senescence. Exp. Cell. Res..

[90] Tonoki A., Kuranaga E., Tomioka T., Hamazaki J., Murata S., Tanaka K., Miura M. (2009). Genetic evidence linking age-dependent attenuation of the 26S proteasome with the aging process. Mol. Cell. Biol..

[91] Babbitt S.E., Kiss A., Deffenbaugh A.E., Chang Y.H., Bailly E., Erdjument-Bromage H., Tempst P., Buranda T., Sklar L.A., Baumler J. (2005). ATP hydrolysis-dependent disassembly of the 26S proteasome is part of the catalytic cycle. Cell.

[92] Thompson D., Hakala K., DeMartino G.N. (2009). Subcomplexes of PA700, the 19 S regulator of the 26 S proteasome, reveal relative roles of AAA subunits in 26 S proteasome assembly and activation and ATPase activity. J. Biol. Chem..

[93] Vilchez D., Boyer L., Morantte I., Lutz M., Merkwirth C., Joyce D., Spencer B., Page L., Masliah E., Berggren W.T. (2012). Increased proteasome activity in human embryonic stem cells is regulated by PSMD11. Nature.

[94] Vilchez D., Morantte I., Liu Z., Douglas P.M., Merkwirth C., Rodrigues A.P., Manning G., Dillin A. (2012). RPN-6 determines *C. elegans* longevity under proteotoxic stress conditions. Nature.

