# Supplementary material for: Base-CP proteasome can serve as a platform for stepwise lid formation
Source: Biosci Rep. 2015 May 19;35(3):e00194. doi: 10.1042/BSR20140173 (PMC4438304; doi:10.1042/BSR20140173)
Supplement: Supplementary data [file bsr035e194ntsadd.pdf]

Supporting Information for:

Base-CP proteasome can serve as a platform for stepwise lid formation

Running title: **Proteasome Lid formation**

Zanlin Yu[*], Nurit Livnat-Levanon[*], Oded Kleifeld[†], Wissam Mansour[*], Mark A. Nakasone[*], Carlos A. Castaneda[‡], Emma K. Dixon[‡], David Fushman[‡], Noa Reis[*], Elah Pick[§], Michael H. Glickman[*, a]

[*] Department of Biology, Technion–Israel Institute of Technology, 32000 Haifa, Israel
[†] Department of Biochemistry & Molecular Biology, Monash University, Clayton, VIC 3800, Australia
[‡] Department of Biology and Environment, University of Haifa at Oranim, Tivon 36006, Israel
[§] Department of Chemistry and Biochemistry, Center for Biomolecular Structure and Organization, University of Maryland, College Park, MD 20742, USA.

[a] Correspondence to: Michael H. Glickman glickman@tx.technion.ac.il

This file contains the following supplementary information

Supplementary Figures 1-3 p. 2

Supplementary Tables 1-2 p. 4

Supplementary Materials and methods p. 5

Supplementary References p. 6

Supplementary Figures

FIGURE S1

a.

b.

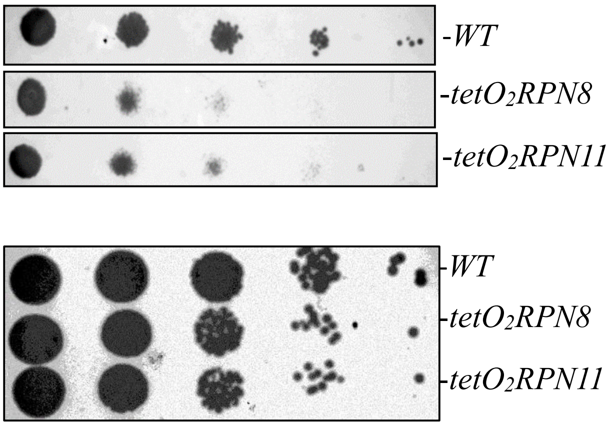

S1. Tetracyclin inducible promoter suppresses its downstream gene expression, resulting in non-observable growth. (a) *tetO*$_2$*Rpn8* and *tetO*$_2$*Rpn11* were serially inoculated in the YPD plates containing 20µg/ml tetracyclin. (b). After treatment of tetracyclin for overnight, the cells as indicated were inoculated to fresh YPD to check the viability.

FIGURE S2

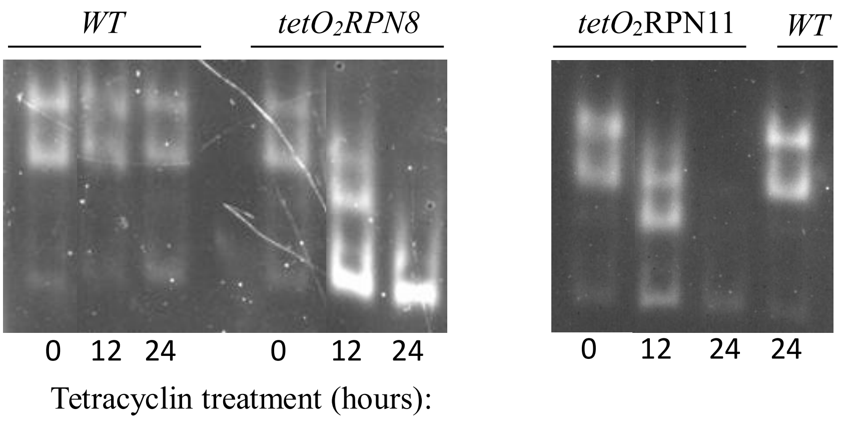

S2. Super-long term treatment of tetracyclin leads to break of proteasome. The yeast strain *tetO₂RPN8* and *tetO₂*RPN11were treated by 20ug/ml tetracyclin for longer period of time as indicated.

FIGURE S3

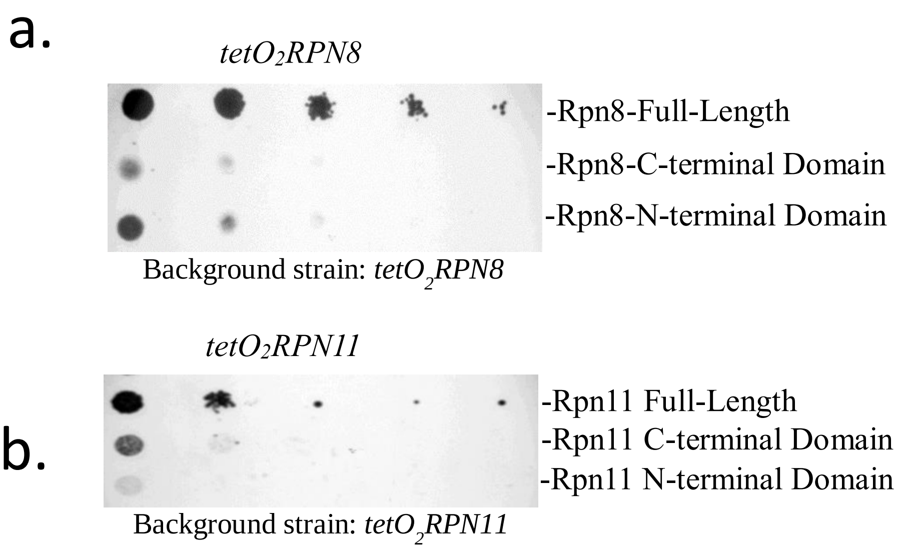

S3. Either N-terminus or C-terminus doesn't rescue the lethality due to the suppression of *RPN8* or *RPN11* (a) Plasmid containing the RPN8 domains as indicated under control of ADH promoter was inducted into the background strain *tetO$_2$RPN8*, the cells were inoculated onto plates containing 20μg/ml tetracyclin in a serial dilution. (b) Plasmid containing the RPN11 domains as indicated under control of ADH promoter was inducted into the background strain *tetO$_2$RPN11*, the cells were inoculated onto plates containing 20μg/ml tetracyclin in a serial dilution.

Supplementary Tables

Yeast strain list

number	Gene	Characteristics	Origin
MY58	WT	*his3ko1; leu2ko0; met15ko0; ura3ko0*	Euroscarf
MY1262	*tetO*$_2$*-Rpn8*	*RPN8*:: kanR-tetO$_7$-TATA-*RPN8*	GE openbiosystems
MY1263	*tetO*$_2$*-Rpn11*	*RPN11*:: kanR-tetO$_7$-TATA-*RPN11*	GE openbiosystems
MY1107	*rpn3-4*	*rpn3*::*rpn3-4*-TRP1	(1)
MY1070	*rpn5-1*	*rpn5*::*rpn5-1*-TRP1	(2)
MY1122	*rpn6-1*	*rpn6*::*rpn6-1*-URA3	(3)
MY1068	*rpn7-3*	*rpn7*::*rpn7-3*-URA3	(4)
MY1123	*rpn9ΔC*	*rpn9*::*rpn9ΔC*-LEU2	(5)
MY1119	*rpn12-1*	*rpn12*::*rpn12-1*-URA3	(1)
MY1268	*rpn11-m1*	*rpn11::rpn11-M1*	(6)
MY1284	*rpn8-1*	*rpn8::rpn8-1-LEU*	This study

The list contains all the plasmid used in this study.

The genetic background of the yeast strains used in this study is based on BY4741

Plasmid list:

Number	Gene	Vector	Origin
M1364	Rpn5, Rpn6, Rpn8, Rpn9, his6-Rpn11	petDuet	(7)
M1335	his6-Rpn11	pQE30	this study
M1388	Rpn8, his6-Rpn11	petDuet	this study
M1398	Rpn5, Rpn8, his6-Rpn11	petDuet	this study
M1397	Rpn5, Rpn8, Rpn9, his6-Rpn11	petDuet	this study
M1400	Rpn6, Rpn8, Rpn9, his6-Rpn11	petDuet	this study
M1386	Rpn5, Rpn6, Rpn8, his6-Rpn11	petDuet	this study
M1403	Rpn5, Rpn6, Rpn9, his6-Rpn11	petDuet	this study
M1109	Rpn11 C-terminus	pQE30	this study
M899	Rpn11 C-terminus	pRS425	(8)

The list contains all the plasmid used in this study.

Methods and Materials in MS/MS analysis

Proteasome resolusion

Whole cell extract from yeast cells was resolved by 4% nondenaturing-PAGE (9). The peptidase activity based on LLVY-AMC tracing was the marker for cutting the gel slices. The native gel slices were modified with 100 mM iodoacetamide in 10 mM ammonium bicarbonate (room temperature for 30min) and trypsinized in 10 mM ammonium bicarbonate containing trypsin [modified trypsin (Promega)] at a 1:50 enzyme-to-substrate ratio, overnight at 37°C.

Mass spectrometry analysis

The resulting tryptic peptides were resolved by reverse-phase chromatography on 0.075 X 200-mm fused silica capillaries (J&W) packed with Reprosil reversed phase material (Dr Maisch GmbH, Germany). The peptides were eluted with linear 65 minutes gradients of 5 to 45% and 15 minutes at 95% acetonitrile with 0.1% formic acid in water at flow rates of 0.25 μl/min. Mass spectrometry was performed by an ion-trap mass spectrometer (Orbitrap, Thermo) in a positive mode using repetitively full MS scan followed by collision induces dissociation (CID) of the 7 most dominant ion selected from the first MS scan.

Database search

The mass spectrometry data was analyzed using the Trans Proteomic Pipeline (TPP) Version 4.3 (10). TPP-processed centroid fragment peak lists in mzXML format were searched against Saccharomyces cerevisiae translations of all systematically named ORFs (Downloaded form SGD). The proteins were supplemented with their corresponding decoy sequences (as described in http://www.matrixscience.com/help/decoy_help.html). The database searches were performed using X! Tandem with k-score plugin through the TPP. Search parameters include: trypsin cleavage specificity with two missed cleavage, cysteine carbamidomethyl as fixed modification, methaionine oxidation and protein N-terminal acetylation as variable modifications, peptide tolerance and MS/MS (11).

Supplementary References

1. Bailly E & Reed SI (1999) Functional characterization of rpn3 uncovers a distinct 19S proteasomal subunit requirement for ubiquitin-dependent proteolysis of cell cycle regulatory proteins in budding yeast. *Mol. Cell. Biol.* 19(10):6872-6890.

2. Isono E, *et al.* (2007) The assembly pathway of the 19S regulatory particle of the yeast 26S proteasome. *Mol Biol Cell* 18(2):569-580.

3. Isono E, Saito N, Kamata N, Saeki Y, & Toh EA (2005) Functional analysis of Rpn6p, a lid component of the 26 S proteasome, using temperature-sensitive rpn6 mutants of the yeast Saccharomyces cerevisiae. *J Biol Chem* 280(8):6537-6547.

4. Isono E, Saeki Y, Yokosawa H, & Toh-e A (2004) Rpn7 Is Required for the Structural Integrity of the 26 S Proteasome of Saccharomyces cerevisiae. *J. Biol. Chem.* 279(26):27168-27176.

5. Takeuchi J, Fujimuro M, Yokosawa H, Tanaka K, & Toh-e A (1999) Rpn9 is required for efficient assembly of the yeast 26S proteasome. *Mol. Cell. Biol.* 10:6575-6584.

6. Rinaldi T, *et al.* (2004) Participation of the proteasomal lid subunit Rpn11 in mitochondrial morphology and function is mapped to a distinct C-terminal domain *Biochem J.* 381(Pt 1):275-285.

7. Lander GC, *et al.* (2012) Complete subunit architecture of the proteasome regulatory particle. *Nature* 482(7384):186-191.

8. Rinaldi T, *et al.* (2008) Dissection of the carboxyl-terminal domain of the proteasomal subunit Rpn11 in maintenance of mitochondrial structure and function. *Mol Biol Cell* 19(3):1022-1031.

9. Livnat-Levanon N, *et al.* (2014) Reversible 26S Proteasome Disassembly upon Mitochondrial Stress. *Cell reports*.

10. Keller A, Eng J, Zhang N, Li XJ, & Aebersold R (2005) A uniform proteomics MS/MS analysis platform utilizing open XML file formats. *Mol Syst Biol* 1:2005 0017.

11. Yu Z, *et al.* (2011) Dual function of Rpn5 in two PCI complexes, the 26S proteasome and COP9 signalosome. *Mol Biol Cell* 22(7):911-920.
